# In Situ Steam-Assisted Synthesis of CTAB-Modified Geopolymer-Based Hectorite for Enhanced Adsorption of Congo Red

**DOI:** 10.3390/gels11110930

**Published:** 2025-11-19

**Authors:** Derui Chen, Chao Sun, Keying Sun, Mingyu Yan, Yang Yang, Hang Jin, Junda Guo, Jingna Jia, Longbin Xu, Xinyu Li

**Affiliations:** 1 College of Engineering, Materials and Chemical Engineering, Yanbian University, Yanji 133002, China; 2023050082@ybu.edu.cn (D.C.); 2024050102@ybu.edu.cn (K.S.); 2024050098@ybu.edu.cn (M.Y.); 2025050044@ybu.edu.cn (Y.Y.); 2025050052@ybu.edu.cn (H.J.); 0000008028@ybu.edu.cn (J.J.); 2 Department of Physics, Jilin University, Changchun 130012, China; sunc344@jlu.edu.cn (C.S.); guojunda@jlu.edu.cn (J.G.); 3 Department of Chemistry, College of Science, Yanbian University, Yanji 133002, China; 4 Department of Polymer Materials & Engineering, College of Engineering, Yanbian University, Yanji 133002, China

**Keywords:** hectorite, steam-assisted method, geopolymer, CTAB, Congo red

## Abstract

For deep purification of wastewater containing anionic dyes. In this study, cetyltrimethylammonium bromide (CTAB)-modified geopolymer-based hectorite was synthesized via a steam-assisted method using depolymerized illite-based geopolymer as the silicon source and CTAB as the modifier, enhancing its adsorption performance for anionic dyes. The product was characterized by methods such as X-ray diffraction, and the effects of parameters such as adsorbent dosage and pH on the adsorption process were investigated. Adsorption experiments revealed that when the CTAB addition was 20%, the adsorption performance for Congo red was optimal (99.79%, 997.92 mg·g^−1^), far superior to that of hectorite without CTAB (66.64%, 666.40 mg·g^−1^). The adsorption process followed pseudo-second-order kinetics and the Langmuir isotherm model. Further comparison of changes before and after adsorption indicated that the adsorption mechanism primarily involved the combined effects of electrostatic interaction and hydrophobic effects. Additionally, after five adsorption–desorption cycles, the material maintained over 92% removal efficiency. By using different geopolymers as silicon sources to prepare CTAB-modified geopolymer-based hectorite, the high universality of this synthesis strategy was confirmed. This study provides a universal, green, and sustainable route for preparing efficient anionic dye adsorption materials and expands the high-value utilization of clay resources.

## 1. Introduction

Human health and survival are closely linked to the quality of the water environment. While the widespread use of synthetic dyes has fueled the growth of the textile industry, it has also led to severe environmental challenges. Wastewater discharged from textile manufacturing contains large amounts of non-biodegradable dyes, which pose a significant threat to ecosystems and human health [1]. Among these, anionic dyes (e.g., Congo Red) are particularly challenging to remove effectively using conventional methods because of their high water solubility and anionic character, which confer high chemical stability [2]. Common treatment techniques, such as photocatalysis [3], biodegradation [4], and membrane filtration [5], have been employed. However, these methods often suffer from limitations including high cost, the potential for secondary pollution, and insufficient removal efficiency for specific pollutants. In contrast, the adsorption method has gained significant attention as a promising alternative due to its low cost and simple operation [6,7]. Therefore, developing efficient and renewable adsorbent materials is key to promoting the practical application of the adsorption method [8,9].

Among the existing adsorption materials, gel materials have been widely used in the field of water remediation [10]. At present, the research on organic gel has been relatively sufficient, while the exploration on inorganic gel is still relatively small [11]. In recent years, significant progress has been made in the research of mineral materials as efficient adsorbents. Hectorite, an inorganic gel material with a unique nanolayered structure, high specific surface area, and abundant surface-active sites, can form a hierarchical “house of cards” network structure in aqueous solutions. This structure provides the necessary sites for adsorption and has demonstrated excellent dye adsorption performance [12,13]. However, due to its electronegativity and hydrophilic nature, hectorite is mostly used for the adsorption of cationic dyes but is ineffective in removing anionic organic dyes [14]. To address this issue, organic modification strategies have been widely employed in recent years for the functionalization of hectorite. For instance, Dai et al. modified hectorite with cetyltrimethylammonium bromide (CTAB) under ethanol-assisted conditions; the combination of the organic modifier with hectorite altered its surface charge distribution, enabling electrostatic attraction with Congo Red and thereby improving adsorption efficiency [15]. Asranudin et al. prepared CTAB-modified hectorite via a one-step method, achieving an adsorption capacity of 164.28 mg·g^−1^ for Methyl Orange (MO) [16]. CTAB modification preserves the structural advantages of hectorite while introducing electrostatic and hydrophobic interactions through the surfactant, enhancing its adsorption capacity for anionic dyes.

However, these conventional modification methods typically require large volumes of organic solvents, which hinders their large-scale application. Furthermore, the synthesis of hectorite itself faces two major challenges. First, the conventional preparation process is inefficient and environmentally burdensome. For instance, industrial-scale hectorite is typically produced through hydrothermal methods using raw materials like SiO_2_ and Mg(OH)_2_ [17]. This process requires several days at elevated temperatures, resulting in high energy consumption, substantial production costs, and the generation of significant wastewater that can cause environmental pollution [18]. Second, the precursors used in hectorite synthesis are often costly. For example, current synthesis routes often rely on expensive precursors such as tetraalkoxysilane [19], dimethyloctylmethoxysilane [20], and organotrialkoxysilane [21]; the use of these reagents substantially increases production costs. These drawbacks collectively hinder the industrial production of hectorite from being cost-effective, scalable, and environmentally sustainable.

To address the aforementioned challenges, the development of novel adsorbent materials that combine high adsorption performance with environmentally friendly characteristics has become a prominent research focus [22]. In recent years, geopolymers have become a highly promising strategic new material with four core advantages: high performance, cost-effective raw materials, energy-saving production, and environmental protection [23]. Geopolymers can be obtained by depolymerizing natural clay. These clays can be subjected to simple pre-treatments like thermal activation or acid washing to remove impurities and enhance their reactivity [24]. Studies on kaolinite [25], attapulgite [26], and rectorite [27] have demonstrated that employing geopolymers as a raw material effectively avoids the high costs of silica production. Furthermore, the steam-assisted method has gained widespread attention as a green and efficient synthesis strategy in the field of material preparation. This method utilizes vapor-phase reactions, effectively eliminating solvent consumption. Although no studies have yet been reported on the modification of hectorite via the steam-assisted method, research on its application to other clays and zeolites provides valuable insights [28,29,30,31].

Therefore, this study selected depolymerized illite-based geopolymer as a silica source and employed cetyltrimethylammonium bromide (CTAB) as a surfactant to synthesize modified hectorite in situ via a steam-assisted method, evaluating its potential as an adsorbent for Congo Red removal. The efficacy of the synthesized materials for removing Congo Red from aqueous solution was evaluated. The influence of CTAB dosage on the material’s morphology and structure was examined. The adsorption process was optimized by investigating key parameters including solution pH, adsorbent dosage, contact time, and temperature. Adsorption kinetics, isotherms, and thermodynamics were analyzed to understand the underlying mechanism. We propose that this synthetic strategy could potentially be extended to other natural clays, such as montmorillonite and kaolinite. Overall, this work demonstrates a simple, low-cost, and green approach for producing high-performance hectorite adsorbents, contributing to the sustainable production of functional materials and the high-value utilization of geopolymers.

## 2. Results and Discussion

### 2.1. Characterization of In Situ Synthesis of CTAB-Modified Geopolymer-Based Hectorite

**Figure 1:**
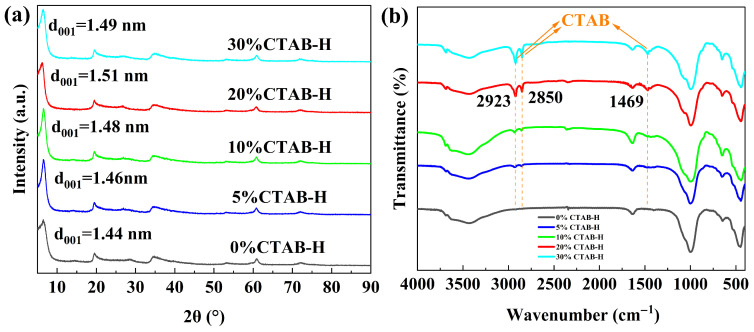
XRD patterns (**a**) and FTIR patterns (**b**) of hectorite and CTAB-modified geopolymer-based hectorite synthesized in situ.

The XRD patterns of the synthesized products with different CTAB loadings were shown in Figure 1a. As illustrated, all patterns exhibit characteristic diffraction peaks at 2θ = 6.44° (001), 19.53° (110, 020), 28.52° (004), 34.78° (130, 200), and 60.86° (060, 330), which are consistent with the typical reflections of hectorite [32]. Comparison of the XRD patterns before and after modification reveals noticeable changes in the (001) diffraction peak. According to Bragg’s law, the basal spacing (d_001_) of the unmodified hectorite sample is calculated to be 1.44 nm. When the CTAB loading increases to 5%, the d_001_ value expands to 1.46 nm. Further increases in CTAB loading to 10% and 20% result in basal spacings of 1.48 nm and 1.51 nm, respectively, indicating the successful intercalation of CTAB molecules into the interlayer galleries of hectorite. When the amount of CTAB added was increased to 30%, the d_001_ value decreased to 1.49 nm. This reduction is attributed to the adverse effect of excessive CTAB on the growth of hectorite, which consequently led to a decrease in the interlayer spacing of the synthesized product. Additionally, the surplus CTAB deposited on the external surface of the hectorite [15,16]. These results demonstrate that the introduction of CTAB leads to an expansion of the basal spacing, confirming the successful insertion of CTAB molecules into the interlayer structure of hectorite [33]. At the same time, all CTAB-modified samples retain XRD patterns similar to that of the unmodified hectorite, indicating that the fundamental layered structure remains highly stable during the modification process [34]. Furthermore, the universality of the in situ synthesis strategy was verified by applying it to montmorillonite and kaolinite as alternative raw materials. As shown in Figures S1 and S2, the CTAB-modified geopolymer-based hectorite samples synthesized from these clays exhibit a series of diffraction peaks that match well with those of the 20%CTAB-H sample. These results indicate that the hectorite and CTAB-modified geopolymer-based hectorite synthesized from either montmorillonite or kaolinite closely resemble those derived from illite, confirming the high universality of the proposed strategy.

The FTIR spectra of the synthesized products with different CTAB loadings were shown in Figure 1b. As observed, the absorption peak at 3442 cm^−1^ is attributed to the stretching vibration of hydroxyl groups (-OH). The characteristic peak at 1646 cm^−1^ arises from the -OH vibration of silanol groups. The absorption peaks at 995 cm^−1^ and 451 cm^−1^ correspond to the stretching and bending vibrations of siloxane groups (Si-O), respectively. The peak at 651 cm^−1^ is assigned to the stretching vibration of Mg(Li)-O(OH) groups. In comparison with the unmodified hectorite (0%CTAB-H), the CTAB-modified samples exhibit two additional characteristic peaks at 2923 cm^−1^ and 2850 cm^−1^, which are attributed to the symmetric C-H vibrations of methyl and methylene groups (-CH_2_-) in CTAB, respectively. The absorption peak appearing around 1469 cm^−1^ originates from the asymmetric bending vibration of N-H bonds in CTAB [35,36]. As the amount of CTAB added increases, the intensity of the characteristic peak of CTAB gradually increases, indicating that CTAB molecules have successfully inserted into the interlayer of hectorite through example exchange, implying the achievement of in situ modification of hectorite [37].

**Figure 2:**
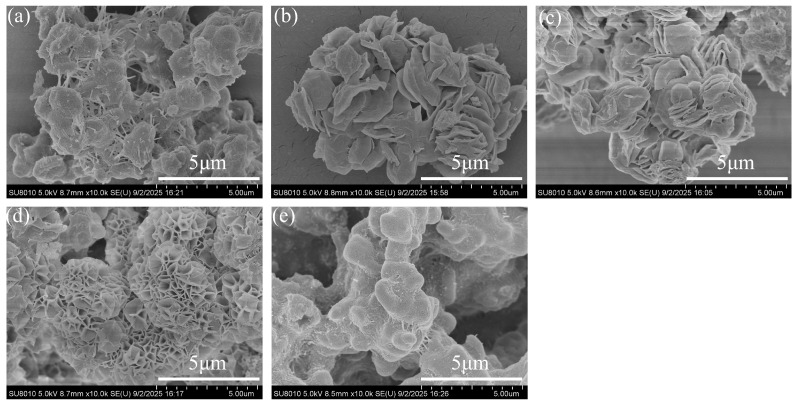
Scanning electron microscopy images of CTAB-modified geopolymer-based hectorite (0%CTAB-H (**a**), 5%CTAB-H (**b**), 10%CTAB-H (**c**), 20%CTAB-H (**d**), 30%CTAB-H (**e**)).

The SEM images of CTAB-modified geopolymer-based hectorite synthesized with different CTAB loadings were shown in Figure 2. The products exhibit distinct microstructures as the CTAB content increases. The unmodified hectorite (0% CTAB) displays smooth crystal surfaces and well-defined grain boundaries. At a CTAB loading of 5%, a slight exfoliation of the layered structure is observed, which can be attributed to the expansion of the interlayer spacing due to CTAB intercalation—consistent with the increased basal spacing revealed by XRD analysis. When the CTAB loading is increased to 10%, the sample morphology becomes denser and begins to transition from a stacked layered structure to a rosette-like morphology. When the amount of CTAB added was increased to 20%, the SEM image (Figure 2d) revealed a regular rose-like structure along with multi-dimensional aggregates, and no impurities were observed. However, when the CTAB addition was further increased to 30%, the layered structures exhibited significant agglomeration. This phenomenon is likely due to the impact of excessive CTAB on the nucleation and growth processes of hectorite, leading to the aggregation between particles.

The SEM images of Hectorite-M, 20%CTAB-H-M, Hectorite-K, and 20%CTAB-H-K were displayed in Figures S3 and S4. The SEM results indicate that both the hectorite and CTAB-modified geopolymer-based hectorite synthesized from either montmorillonite or kaolinite exhibit typical layered structures, which is consistent with the XRD findings. Furthermore, the hectorite particles derived from kaolinite appear larger in size and exhibit more distinct layer thickness, which may be attributed to residual, incompletely disrupted aluminum-oxygen octahedral frameworks present in the depolymerized kaolinite product. The combined evidence from morphology and structure strongly demonstrates that using geopolymers derived from the depolymerization of these two distinct natural clays as raw materials not only enables the production of high-purity silica but also enables the successful synthesis of CTAB-modified geopolymer-based hectorite via the steam-assisted method.

**Figure 3:**
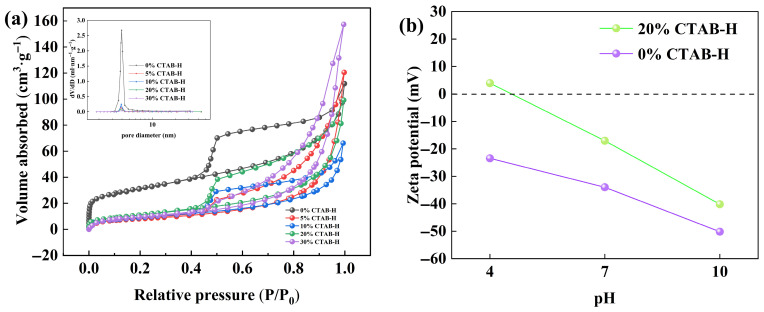
Nitrogen adsorption–desorption curves, pore size distribution (**a**), and zeta point map of hectorite and CTAB-modified geopolymer-based hectorite (**b**).

The N_2_ adsorption–desorption isotherms and pore size distributions of the CTAB-modified geopolymer-based hectorite samples were presented in Figure 3a. As shown, all samples exhibit type IV isotherms with H_3_-type hysteresis loops, indicating the presence of typical mesoporous structures [38]. The specific surface area of the unmodified hectorite (0% CTAB) was measured to be 108.11 m^2^·g^−1^. In contrast, the CTAB-modified samples showed a decrease in specific surface area, with values of 28.93 m^2^·g^−1^ for 5%CTAB-H, 33.17 m^2^·g^−1^ for 10%CTAB-H, 41.77 m^2^·g^−1^ for 20%CTAB-H, and 34.69 m^2^·g^−1^ for 30%CTAB-H. This reduction may be attributed to the aggregation of positively charged CTAB molecules on the surface and within the interlayer spaces of hectorite, leading to partial pore blockage [15]. As the CTAB loading increased from 5% to 20%, a slight upward trend in specific surface area was observed, likely due to structural expansion induced by CTAB intercalation. However, when the CTAB loading reached 30%, the specific surface area decreased again, which can be attributed to agglomeration caused by excessive surfactant. Pore size distributions analyzed by the Barrett–Joyner–Halenda (BJH) method revealed a noticeable increase in the average pore size after CTAB modification. The unmodified hectorite exhibited an average pore size of 5.50 nm, while the CTAB-modified samples showed increased average pore sizes of 11.43 nm (5%CTAB-H), 13.23 nm (10%CTAB-H), 22.86 nm (20%CTAB-H), and 26.69 nm (30%CTAB-H). These results demonstrate that the incorporation of the organic modifier promotes the formation of larger mesopores, highlighting the role of CTAB intercalation in tuning the porous structure of hectorite [15,39].

The zeta potentials of 0%CTAB-H and 20%CTAB-H at different pH values are compared in Figure 3b. A key observation is that the modification with CTAB significantly reduced the negative surface charge across the entire pH range. Most notably, 20%CTAB-H exhibited a charge reversal from negative to positive (+3.9 mV) under acidic conditions (pH = 4), whereas the unmodified 0%CTAB-H remained negatively charged (−23.43 mV). This difference in surface potential provides a key driving force for the adsorption process, enabling the modified material to efficiently remove anionic dyes via electrostatic interactions [15].

### 2.2. Effect of CTAB Loading on Adsorption Performance

**Figure 4:**
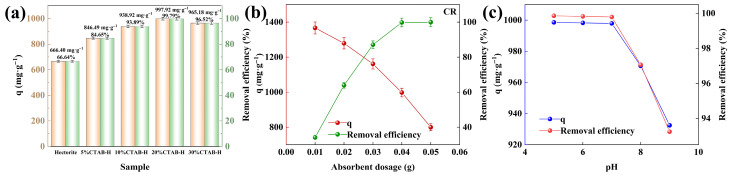
The influence of CTAB content on adsorption (**a**), the influence of adsorbent dosage on adsorption (**b**), the influence of pH on adsorption (**c**).

The effect of CTAB content on the adsorption capacity of the synthesized materials was illustrated in Figure 4a. The adsorption tests were conducted under the following conditions: temperature = 318 K, 100 mL of Congo Red solution with an initial concentration of 400 mg·L^−1^, and adsorbent dosage = 0.04 g. As shown, the unmodified hectorite (0%CTAB-H) exhibits an adsorption capacity of 666.40 mg·g^−1^, corresponding to a Congo Red removal rate of 66.64%. With the addition of 5% CTAB, the removal rate increases to 84.65%, and the adsorption capacity rises to 846.49 mg·g^−1^. When the CTAB loading reaches 10%, the removal rate further improves to 93.89%, with an adsorption capacity of 938.92 mg·g^−1^. At 20% CTAB loading, the sample demonstrates the highest adsorption capacity (997.92 mg·g^−1^) and removal rate (99.79%). The unmodified hectorite possesses a hydrophilic surface that tends to adsorb water molecules, whereas CTAB modification transforms it into a hydrophobic surface, promoting stronger interaction with Congo Red molecules [40]. These results indicate that the incorporation of CTAB significantly enhances the adsorption performance of hectorite toward Congo Red. The continuous increase in adsorption capacity with CTAB loading from 5% to 20% suggests effective modification within this range. However, when the CTAB loading is further increased to 30%, both the adsorption capacity and removal efficiency decline. This can be attributed to the excessive intercalation and surface deposition of CTAB molecules, which may cover active sites or block pore structures [41]. The saturation adsorption capacity achieved with 20% CTAB-H can be attributed to the synergistic effects of multiple mechanisms during the adsorption process, where the optimal CTAB addition (20%) precisely maximizes these cooperative interactions.

### 2.3. Effect of Adsorbent Dosage on Adsorption Performance

The influence of adsorbent dosage on the adsorption of Congo Red (CR) was illustrated in Figure 4b. The experiments were conducted under fixed conditions: initial CR concentration C_0_ = 400 mg·L^−1^, temperature = 318 K, and pH = 7. As shown, increasing the adsorbent dosage from 0.01 g to 0.04 g significantly enhanced the CR removal rate from 34.17% to 99.79%. This improvement is primarily attributed to the increased availability of active sites, which promotes the capture and removal of pollutant molecules. In contrast, the adsorption capacity exhibited a decreasing trend with higher adsorbent loadings. The maximum adsorption capacity of 1366.85 mg·g^−1^ was achieved at a dosage of 0.01 g, while further increasing the dosage to 0.05 g resulted in a reduced capacity of 799.70 mg·g^−1^. These results indicate that at lower dosages, the adsorbent particles remain well-dispersed with fully exposed active sites, leading to higher adsorption efficiency per unit mass. However, at higher dosages, particle aggregation likely occurs, resulting in the shielding or mutual blockage of active sites and consequently a decline in adsorption capacity [42,43].

### 2.4. Effect of pH on Adsorption Performance

The influence of pH on the adsorption behavior of the 20%CTAB-H sample was illustrated in Figure 4c. The experiments were conducted under the following conditions: initial Congo Red concentration C_0_ = 400 mg·L^−1^, temperature = 318 K, and adsorbent dosage = 0.04 g. As shown in the figure, 20%CTAB-H exhibits high Congo red removal efficiency and adsorption capacity within the pH range of 5 to 9, with removal rates exceeding 90% and adsorption capacities exceeding 920 mg·g^−1^. Indicating that 20%CTAB-H exhibits good adsorption stability over a wide pH range. Further analysis revealed that under acidic and neutral conditions (pH 5–7), the removal efficiency of Congo Red is relatively similar; Under alkaline conditions (pH > 7), the removal rate slightly decreases. When the solution is under acidic conditions, combined with the zeta potential diagram in Figure 3b, the adsorbent surface is positively charged, which facilitates the adsorption of Congo red (whose sulfonic acid ions are negatively charged) through electrostatic interactions. As the pH increases to the alkaline range, the surface of the adsorbent gradually becomes negatively charged, resulting in electrostatic repulsion with the similarly negatively charged Congo red, leading to a decrease in adsorption capacity. In addition, high concentrations of OH^−^ ions in alkaline media compete with Congo red for limited surface adsorption sites, further inhibiting the adsorption process. The adsorption performance under acidic and neutral conditions is similar, suggesting that the adsorption system may be dominated by physical adsorption mechanism [16]. In alkaline environments, the increase in negative charge on the surface of materials leads to electrostatic repulsion, resulting in a slight decrease in adsorption performance. However, the small decrease in adsorption capacity suggests that electrostatic interactions are not the only reason for Congo red adsorption. This behavior is consistent with the conclusions of multiple studies that physical adsorption dominant systems maintain high adsorption capacity over a wide pH range [44,45]. Therefore, this study chose to conduct subsequent experiments under pH = 7.0 conditions.

### 2.5. Effect of Contact Time and Adsorption Kinetics

**Figure 5:**
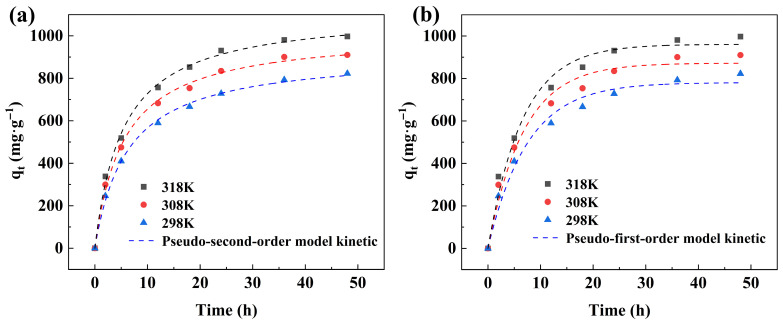
Kinetic fitting adsorption by 20%CTAB-H: Pseudo-second-order kinetic model (**a**), pseudo-first-order kinetic model (**b**).

The kinetic fitting of Congo Red adsorption onto 20%CTAB-H at different temperatures was shown in Figure 5. The experiments were conducted under the following initial conditions: C_0_ = 400 mg·L^−1^, adsorbent dosage = 0.04 g, and pH = 7. As observed, the adsorption capacity increases with contact time within the range of 0–48 h. During the initial 0–12 h period, the adsorption capacity rises rapidly, which can be attributed to the high probability of contact between the active sites of the adsorbent and the pollutant molecules. Subsequently, the rate of adsorption gradually slows down, and the saturation capacity is essentially reached after 36 h. Further extension of the adsorption time does not lead to significant changes in adsorption capacity, indicating that most active sites on the sample have been occupied by Congo Red molecules. The specific parameters of the corresponding kinetic models are summarized in Table 1. As listed, the correlation coefficients (R^2^) of the pseudo-second-order model for 20%CTAB-H are 0.997, 0.997, and 0.998 at the tested temperatures, while those of the pseudo-first-order model are 0.982, 0.978, and 0.979, respectively. The higher R^2^ values of the pseudo-second-order model indicate that it more accurately describes the adsorption process. Furthermore, the theoretical equilibrium adsorption capacities calculated by the pseudo-second-order model are in close agreement with the experimental values, confirming its applicability.

### 2.6. Effect of Initial Concentration and Adsorption Isotherms

**Figure 6:**
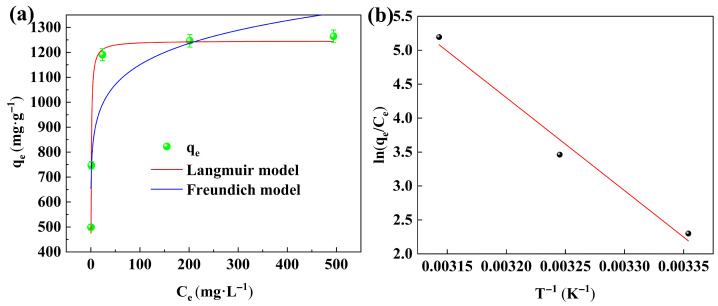
Langmuir and Freundlich models (**a**); van’t Hoff plots for 20%CTAB-H (**b**).

The fitting results of the Langmuir and Freundlich models were presented in Figure 6a, and all corresponding isotherm parameters were summarized in Table 2. The experiments were performed under the following initial conditions: temperature = 318 K, adsorbent dosage = 0.04 g, and pH = 7. The results indicate that the adsorption capacity of Congo Red increases with rising initial concentration. This trend can be attributed to the enhanced mass transfer driving force resulting from the higher concentration gradient, which promotes the adsorption process. As listed in Table 2, the Langmuir model exhibits a higher correlation coefficient (R^2^ = 0.995) compared to the Freundlich model (R^2^ = 0.798), indicating that the Langmuir model provides a more accurate description of the adsorption process. This further suggests that the adsorption of Congo Red onto 20%CTAB-H occurs primarily via monolayer coverage. The maximum adsorption capacity calculated from the Langmuir model is 1246.13 mg·g^−1^. Compared with other adsorbent materials reported in the literature (Table 3), the 20%CTAB-H sample synthesized in this work demonstrates superior adsorption performance.

### 2.7. Effect of Temperature and Thermodynamics on Adsorption Performance

The variation in adsorption capacity of 20%CTAB-H at 298 K, 308 K, and 318 K was shown in Figure 5. As illustrated, the adsorption capacity gradually increases with rising temperature. To further understand the adsorption behavior of Congo Red on 20%CTAB-H, thermodynamic analysis was performed, and the corresponding parameters are summarized in Table 4. Figure 6b presents the van ’t Hoff plots for 20%CTAB-H. As listed in Table 4, the positive value of ΔH indicates that the adsorption process is endothermic and increasing the temperature favors adsorption. The negative ΔG values suggest that the adsorption occurs spontaneously without requiring external energy. The fact that ΔG falls within the range of –20 to 0 kJ/mol implies that the adsorption is predominantly a physical process [40]. Furthermore, the positive ΔS value reflects an increase in the unpredictability of interactions between the adsorbent and Congo Red molecules at higher temperatures [15].

### 2.8. Regeneration Performance of the Adsorbent

**Figure 7:**
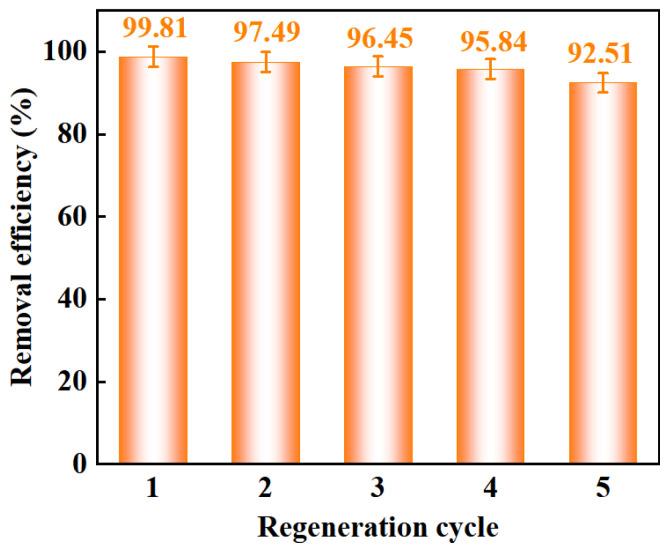
Reusable performance of 20%CTAB-H (Volume of solution: 100 mL, Initial concentration: 400 mg·L^−1^, Adsorbent dosage: 0.04 g, Adsorption time: 36 h, Temp: 318 K, pH = 7).

The regeneration capability of an adsorbent is of significant importance for its practical application in wastewater treatment. As shown in Figure 7, after five consecutive adsorption–desorption cycles, the Congo Red removal rate decreased from the initial 99.81% to 92.51%. These results clearly demonstrate that 20%CTAB-H not only allows effective desorption of Congo Red but also maintains excellent reusability, highlighting its considerable potential for the removal of dyes from contaminated water.

### 2.9. Adsorption Mechanism of Congo Red

**Figure 8:**
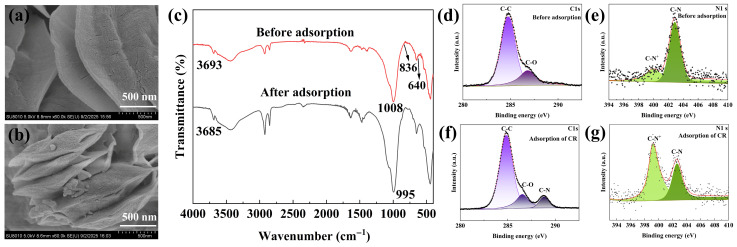
SEM images of 20%CTAB-H before adsorption (**a**) and after adsorption of CR (**b**), FTIR spectra of 20%CTAB-H before and after adsorption of CR (**c**), XPS spectra of C 1s (**d**,**f**) and N 1s (**e**,**g**) before and after adsorption of 20%CTAB-H.

The SEM images of 20%CTAB-H before and after Congo Red (CR) adsorption were presented in Figure 8a,b, respectively. The images reveal that the layered structure of the material remains intact after adsorption, demonstrating its structural stability. The FTIR spectra of the samples before and after adsorption are shown in Figure 8c. After CR adsorption, the characteristic peak at 3693 cm^−1^ shifts to 3685 cm^−1^, which corresponds to the stretching vibration of the N–H bond in CR. The peak at 1008 cm^−1^ shifts to 995 cm^−1^, indicating the presence of the -SO_3_^−^ group from CR, while the peaks at 836 cm^−1^ and 640 cm^−1^ are associated with benzene ring vibrations and C–S bonds in CR, respectively [53]. These FTIR results suggest that the adsorption of CR onto 20%CTAB-H occurs primarily through physical interactions, with possible mechanisms including electrostatic attraction between the NH_3_^+^ groups of CTAB and the -SO_3_^−^ groups of CR, as well as hydrophobic interactions. The C 1s and N 1s XPS spectra before and after adsorption are displayed in Figure 8d–g. As shown in Figure 8d,f, the C 1s peaks are mainly composed of C–C and C–O bonds both before and after adsorption. However, the appearance of a C–N peak after adsorption indicates the physical adsorption of CR [15]. Figure 8e,g show the N 1s XPS spectra before and after CR adsorption, respectively. Before adsorption, the two N 1s peaks originate from the quaternary ammonium groups of CTAB, with binding energies of 400.0 eV for the C–N^+^ component and 402.9 eV for the C–N component [54]. After CR adsorption, the corresponding binding energies shift to 399.1 eV and 402.5 eV, respectively. The intensity of the C–N^+^ peak increases significantly, while that of the C–N peak decreases. This suggests that the quaternary ammonium groups in CTAB play a dominant role in binding the dye molecules, with partial conversion of C–N to C–N^+^ accompanied by negative shifts in binding energy. The XPS results provide direct evidence for the effective capture of anionic dye molecules by the positively charged sites on the material surface, further confirming that electrostatic interaction is the dominant mechanism in the adsorption process [55].

**Figure 9:**
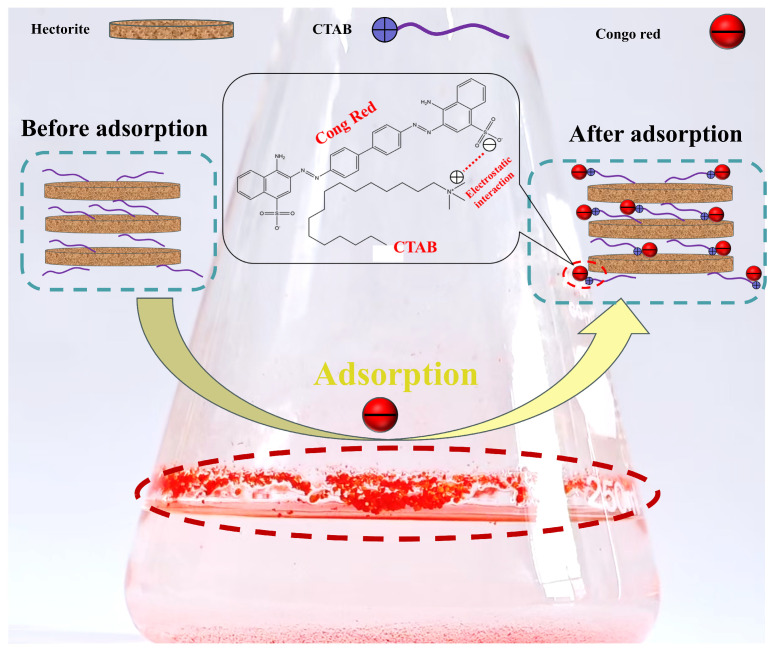
Schematic diagram of the interaction mechanism between 20%CTAB-H and Congo red.

Figure 9 illustrates the proposed adsorption mechanism of Congo Red (CR) on the in situ synthesized CTAB-modified geopolymer-based hectorite (20%CTAB-H). As a cationic surfactant, CTAB is dispersed on the surface and within the interlayers of 20%CTAB-H through electrostatic interactions and interlayer ion exchange. The Congo Red molecule, which contains sulfonate anions, can be attracted to the modified hectorite via electrostatic attraction. In addition, CTAB alters the hydrophilic nature of the original hectorite and introduces alkyl functional groups, enabling hydrophobic interactions with CR and thereby increasing the number of hydrophobic adsorption sites [56]. Therefore, the adsorption mechanism of CR on the modified material can be attributed to the combined effects of electrostatic attraction and hydrophobic interaction.

## 3. Conclusions

This study developed a novel green “synthesis and modification” approach that utilizes geopolymers derived from depolymerized natural clay as a low-cost silica source in a steam-assisted system. By introducing CTAB directly during the nucleation and growth stages of hectorite crystals, we achieved the in situ synthesis of organically modified geopolymer-based hectorite with high anionic dye adsorption performance. Structural evolution of the products under different CTAB loadings was characterized by XRD, FTIR, and SEM analyses. When no CTAB was added, the product consisted of hectorite. Within a CTAB loading range of 0–20%, increasing CTAB content led to its intercalation into the hectorite interlayers, progressively expanding the interlayer spacing. The morphology of hectorite evolved from an initial layered structure to eventual exfoliation, finally forming a rose-petal-like morphology. Meanwhile, CTAB incorporation effectively neutralized the negative surface charge of hectorite and even induced charge reversal from negative to positive under acidic conditions (20%CTAB-H). Adsorption experiments demonstrated that 20%CTAB-H effectively removed Congo Red, with the adsorption behavior following the Langmuir isotherm and pseudo-second-order kinetic models. The maximum adsorption capacity calculated by the Langmuir model reached 1246.13 mg·g^−1^ (20%CTAB-H). Thermodynamic analysis confirmed a spontaneous and endothermic process. After five adsorption–desorption cycles, the removal efficiency remained above 92%. Mechanism analysis indicated that the adsorption was physically driven, primarily through electrostatic and hydrophobic interactions. Therefore, the synthesized material shows promising potential for removing Congo Red from wastewater.

## 4. Materials and Methods

### 4.1. Materials

Illite (collected from Changbai Mountain, Yanji, China), Montmorillonite (Sinopharm Group Chemical Reagent Co., Ltd. Beijing, China), Kaolin (Maoming, China), K_2_CO_3_ (Tianjin Branch Miou Technology Co., Ltd. Tianjin, China), H_2_SO_4_ (Tianjin Branch Miou Technology Co., Ltd. Tianjin, China), HCl (Tianjin Branch Miou Technology Co., Ltd. Tianjin, China), Mg(OH)_2_ (Tianjin Branch Miou Technology Co., Ltd. Tianjin, China), LiOH·H_2_O (Tianjin Branch Miou Technology Co., Ltd. Tianjin, China), NH_3_·H_2_O (Tianjin Branch Miou Technology Co., Ltd. Tianjin, China), Cetyltrimethylammonium bromide (CTAB) (Tianjin Branch Miou Technology Co., Ltd. Tianjin, China), Congo Red (CR) (Tianjin Branch Miou Technology Co., Ltd. Tianjin, China), All chemicals were used as received without further purification.

### 4.2. Synthesis of CTAB-Modified Geopolymer-Based Hectorite

Preparation of Illite Silica Residue (I-SR): Following the procedure reported in the literature, the natural illite mineral was subjected to pretreatment steps, including calcination and acid leaching to obtain the activated silica residue, denoted as geopolymer [32].

**Figure 10:**
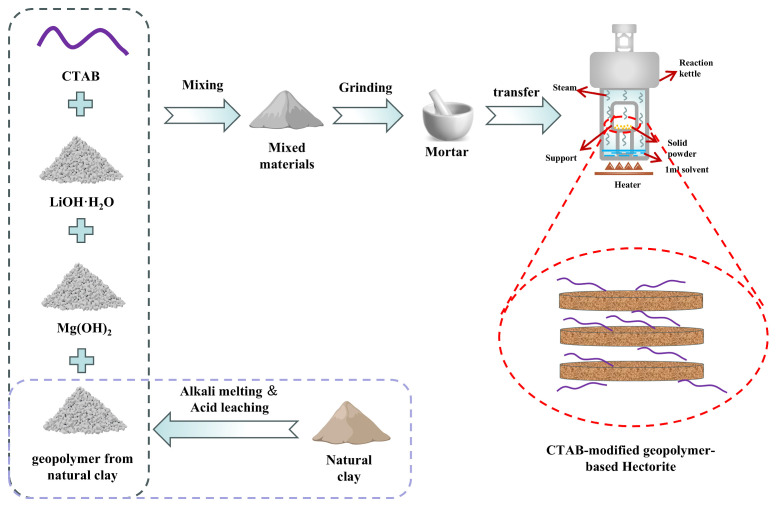
Synthesis flowchart.

In situ synthesis of CTAB-modified geopolymer-based hectorite: Weigh CTAB powder with different addition ratios (based on the total mixture) and directly mix it with I-SR, Mg(OH)_2_, and LiOH·H_2_O. Among them, I-SR, Mg (OH)_2_, and LiOH·H_2_O are 1 g, 0.58 g, and 0.21 g, respectively. The mass ratios of CTAB to the total mixture are 0%, 5%, 10%, 20%, and 30%, respectively. Table S1 shows the specific synthesis parameters. The mixture was placed on a support (to achieve solid–liquid separation) and transferred into a 100 mL Teflon-lined stainless steel autoclave containing only 1 mL of ammonia solution. The reaction was carried out at 200 °C for 24 h. After crystallization, the solid product was collected by centrifugation, washed with deionized water and ethanol, and dried. The resulting samples were denoted as 0%CTAB-H, 5%CTAB-H, 10%CTAB-H, 20%CTAB-H, and 30%CTAB-H, respectively. When montmorillonite was used as the raw material with CTAB additions of 0% and 20%, the corresponding products were labeled as Hectorite-M and 20%CTAB-H-M. Similarly, when kaolinite was employed as the silica source under the same CTAB dosage conditions, the synthesized materials were designated as Hectorite-K and 20%CTAB-H-K. The synthesis flowchart is shown in Figure 10.

### 4.3. Characterization

The prepared samples’ crystal structure and chemical compositions were obtained via powder X-ray diffraction (XRD, Aeris, PANalytical, Almelo, The Netherlands) pattern. According to calculations based on Bragg’s law (2dsinθ = nλ), this directly reflects the variation in the interlayer spacing of the synthesized products [16]. The textural properties were probed by N_2_ adsorption–desorption measurements at −196 °C (BET, 3H-2000, BeiShiDe, Beijing, China). The framework structure of the samples was determined by Fourier Transform Infrared Spectroscopy (FTIR), using a Fourier Transform Infrared Spectrum Analyzer (FTIR-650, Gangdong Technology, Tianjin, China). The X-ray photoelectron spectrometer (Nexsa G2, Thermo Scientific, Waltham, MA, USA) was used to obtain the elements and chemical valence states of the samples. The crystal morphology and size were determined by using scanning electron microscopy (SEM; SU8010, Hitachi, Tokyo, Japan). The concentrations of CR were analyzed via UV–vis spectroscopy (Shimadzu UV-2600, Kyoto, Japan) at a wavelength of 497 nm.

### 4.4. Adsorption Performance of the Synthesized CTAB-Modified Geopolymer-Based Hectorite

Batch adsorption tests were conducted in 250 mL conical flasks using a thermostatic shaker set at a constant stirring speed of 270 rpm. The adsorbent used was synthesized hectorite (labeled as 20%CTAB-H unless noted otherwise). Parameters including adsorbent dosage, solution pH, contact time, and temperature were systematically studied to evaluate their influence on Congo Red (CR) adsorption.

The concentration of CR was analyzed with a UV–vis spectrophotometer at a wavelength of 497 nm. A standard calibration curve, established through linear regression of CR reference solutions, was used for quantification. All tests were carried out in triplicate, and average values are reported, with error bars indicating ±1 standard deviation from three independent measurements.

The equilibrium adsorption capacities (q, mg·g^−1^) of CR on the adsorbent were calculated using Equation (1), while the removal efficiencies (R, %) for CR were determined by Equation (2):

(1) $$q=\frac{(C_{0}-C_{t})V}{m}$$

(2) $$R=\frac{C_{0}-C_{t}}{C_{0}}$$

where R is the pollutant removal efficiency (%), q is the adsorption capacity (mg·g^−1^), C_0_ and C_t_ are the concentration before and after adsorption (mg·L^−1^), V is the solution volume (L), m is the weight of the adsorbent (g).

The pseudo-first-order and pseudo-second-order kinetic models were used to explain the adsorption dynamics according to Equations (3) and (4):

(3) $$q_{t}=q_{e}(1-e^{-K_{1}t})$$

(4) $$q_{t}=\frac{q_{e}^{2}K_{2}t}{1+q_{e}K_{2}t}$$

where q_e_ and q_t_ are the adsorption capacities at equilibrium and at time t (mg·g^−1^), K_1_ (min^−1^) and K_2_ (mg·g^−1^·min^−1^) are the pseudo-first-order kinetics, pseudo-second-order kinetics, respectively.

The Langmuir and the Freundlich isothermal models were used to evaluate the adsorption process using Equations (5) and (6):

(5) $$q_{e}=\frac{K_{L}q_{m}C_{e}}{1+K_{L}C_{e}}$$

(6) $$q_{e}=K_{F}C_{e}^{\frac{1}{n}}$$

where C_e_ is the equilibrium concentration (mg·L^−1^), q_m_ is the maximum adsorption capacity (mg·g^−1^), K_L_ is the Langmuir constant (L·mg^−1^), K_F_ is the Freundlich constant (mg^1-n−1^·L^n−1^·g^−1^), n is the Freundlich linearity index.

Thermodynamic parameters of Gibbs free energy (ΔG^0^, kJ·mol^−1^), enthalpy (ΔH^0^, kJ·mol^−1^), and entropy (ΔS^0^, kJ·mol^−1^·K^−1^) can be used to analyze the thermodynamics based on the following Equations (7) and (8):

(7) $$\Delta G^{0}=\Delta H-T\Delta S^{0}$$

(8) $$lnK_{d}=\frac{\Delta S^{0}}{R}-\frac{\Delta H^{0}}{RT}$$

where R is 8.314 (J·mol^−1^·K^−1^), K_d_ represents the thermodynamic constant, the value of which is equal to that of the Langmuir equilibrium constant.

Following the kinetics adsorption experiment, the spent adsorbent was subjected to a desorption process in 0.1 mol·L^−1^ NaOH at 298 K and 270 rpm. This step was repeated multiple times until Congo Red (CR) was fully removed. The mixture was then centrifuged, and the solid was sequentially rinsed with 0.1 mol·L^−1^ HCl and distilled water. The regenerated hectorite was subsequently applied to the next adsorption cycle under identical conditions. This entire desorption-adsorption procedure was repeated for five cycles. The CR concentration was monitored by UV–vis spectroscopy, and the removal efficiency for each cycle was determined using Equation (2).

## Figures and Tables

**Table 1:** Kinetics model for the adsorption of CR on 20%CTAB-H.

Temp (K)	Adsorbate	Pseudo-First-Order Model			Pseudo-Second-Order Model		
		q_e·cal_ (mg·g^−1^)	K_1_ (min^−1^)	R^2^	q_e·cal_ (mg·g^−1^)	K_2_ (g·mg^−1^·min^−1^)	R^2^
298	CR	781.06	0.134	0.979	918.54	1.63	0.998
308	CR	872.49	0.147	0.978	1015.87	1.79	0.997
318	CR	961.05	0.152	0.981	1115.25	1.75	0.997

**Table 2:** Isotherm modeling parameters for the adsorption of CR by 20%CTAB-H.

Adsorbate	Langmuir Constants			Freundlich Constants		
	q_m_ (mg·g^−1^)	K_L_ (L·mg^−1^)	R^2^	K_F_ (mg^1−n−1^·L^n−1^·g^−1^)	n^−1^	R^2^
CR	1246.13	1.457	0.995	715.13	0.103	0.798

**Table 3:** Maximum adsorption capacity of different adsorbents for CR.

Pollutant	Adsorbent	q_e_ (mg·g^−1^)	Ref
CR	CTAB-La@D	451.1	[15]
	7% Fe_3_O_4_/kaolinite	138.5	[46]
	Crosslinked chitosan immobilized bentonite	787.4	[47]
	Carboxymethyl chitosan hybrid montmorillonite	81.8	[48]
	Chitosan modified hybrid Azadirachta indica leaves powder-Kaolinite	104.6	[49]
	CTAB-modified orange peel biochar	210	[50]
	ZnCuCr-TpIm metal–organic framework	325	[51]
	chitosan-Laponite	390.3	[45]
	bentonite/chitosan@cobalt oxide	303.0	[52]
	20%CTAB-H	1246.13	This work

**Table 4:** Thermodynamic Parameters for CR Adsorption.

Adsorbate	∆H^o^ (J·mol^−1^)	∆S^o^ (J·mol^−1^·K^−1^)	∆G^o^ (kJ·mol^−1^)		
			298 K	308 K	318 K
CR	113.90	0.40	−5.70	−8.87	−13.74

## Data Availability

The original contributions presented in this study are included in the article/Supplementary Materials. Further inquiries can be directed to the corresponding authors.

## Supplementary Materials

The following supporting information can be downloaded at: https://www.mdpi.com/article/10.3390/gels11110930/s1, Table S1. Reaction parameters. Figure S1. XRD of CTAB-modified geopolymer-based hectorite synthesized from depolymerized montmorillonite geopolymer. Figure S2. XRD of CTAB-modified geopolymer-based hectorite synthesized from depolymerized kaolin geopolymer. Figure S3. SEM image of 0%CTAB-H-M (a) and 20%CTAB-H-M synthesized by depolymerized montmorillonite geopolymer (b). Figure S4. SEM image of 0%CTAB-H-M (a) and 20%CTAB-H-M synthesized by depolymerized kaolin geopolymer (b).
