# Supplementary material for: In Situ Steam-Assisted Synthesis of CTAB-Modified Geopolymer-Based Hectorite for Enhanced Adsorption of Congo Red
Source: Gels. 2025 Nov 19;11(11):930. doi: 10.3390/gels11110930 (PMC12651987; doi:10.3390/gels11110930)
Supplement: Supplementary file 1 [file gels-11-00930-s001.zip › gels-3977580-supplementary.pdf]

Supplementary Material

In Situ Steam-Assisted Synthesis of CTAB-Modified Geopolymer-Based Hectorite for Enhanced Adsorption of Congo Red

Derui Chen [1,†], Chao Sun [2,†], Keying Sun [1], Mingyu Yan [1], Yang Yang [1], Hang Jin [1], Junda Guo [2], Jingna Jia [1,3,4], Longbin Xu [1,4,*] and Xinyu Li [1,3,4,*]

[1] College of Engineering, Materials and Chemical Engineering, Yanbian University, Yanji 133002, China; 2023050082@ybu.edu.cn (D.C.); 2024050102@ybu.edu.cn (K.S.); 2024050098@ybu.edu.cn (M.Y.); 2025050044@ybu.edu.cn (Y.Y.); 2025050052@ybu.edu.cn (H.J.); 0000008028@ybu.edu.cn (J.J.)

[2] Department of Physics, Jilin University, Changchun 130012, China; sunc344@jlu.edu.cn (C.S.); guojunda@jlu.edu.cn (J.G.)

[3] Department of Chemistry, College of Science, Yanbian University, Yanji 133002, China

[4] Department of Polymer Materials & Engineering, College of Engineering, Yanbian University, Yanji 133002, China

* Correspondence: longbinx@ybu.edu.cn (L.X.); xinyuli@ybu.edu.cn (X.L.)

† These authors contributed equally to this work.

Figure and table captions

Table S1. Reaction parameters.

Figure S1. XRD of CTAB-modified geopolymer-based hectorite synthesized from depolymerized montmorillonite geopolymer.

Figure S2. XRD of CTAB-modified geopolymer-based hectorite synthesized fromdepolymerized kaolin geopolymer.

Figure S3. SEM image of 0%CTAB-H-M (a) and 20%CTAB-H-M synthesized by depolymerized montmorillonite geopolymer (b).

Figure S4. SEM image of 0%CTAB-H-M (a) and 20%CTAB-H-M synthesized by depolymerized kaolin geopolymer (b).

Table S1. Reaction parameters.

Sample	The amount of CTAB added (g)	Starting compositions			Temp (K)	Time (h)
		Geopolymers (g)	$Mg(OH)_2$ (g)	$LiOH{\cdot}H_2O$ (g)		
0%CTAB-H	0	1	0.58	0.21	473	24
5%CTAB-H	0.09	1	0.58	0.21	473	24
10%CTAB-H	0.18	1	0.58	0.21	473	24
20%CTAB-H	0.36	1	0.58	0.21	473	24
30%CTAB-H	0.54	1	0.58	0.21	473	24

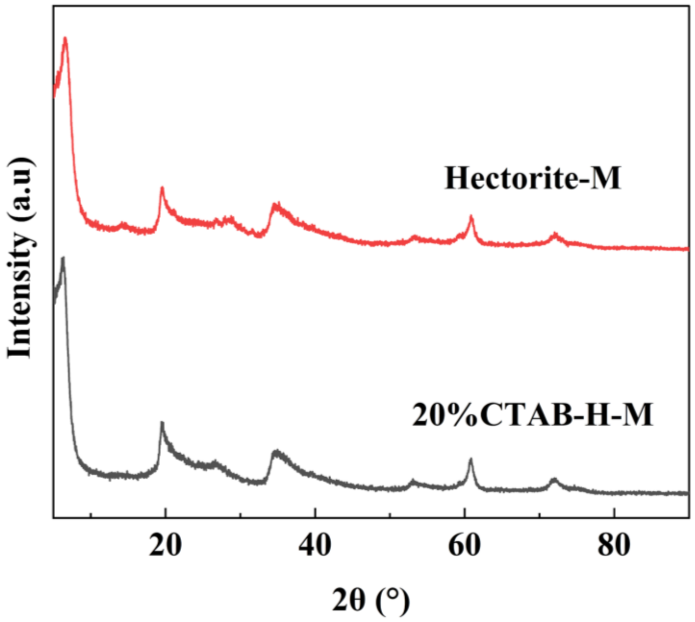

Figure S1. XRD of CTAB-modified geopolymer-based hectorite synthesized from depolymerized montmorillonite geopolymer.

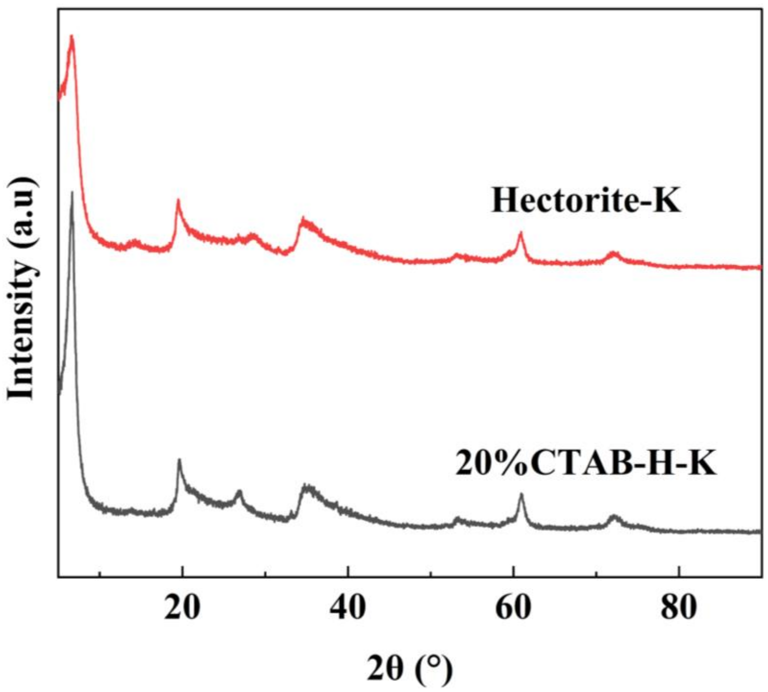

Figure S2. XRD of CTAB-modified geopolymer-based hectorite synthesized from depolymerized kaolin geopolymer.

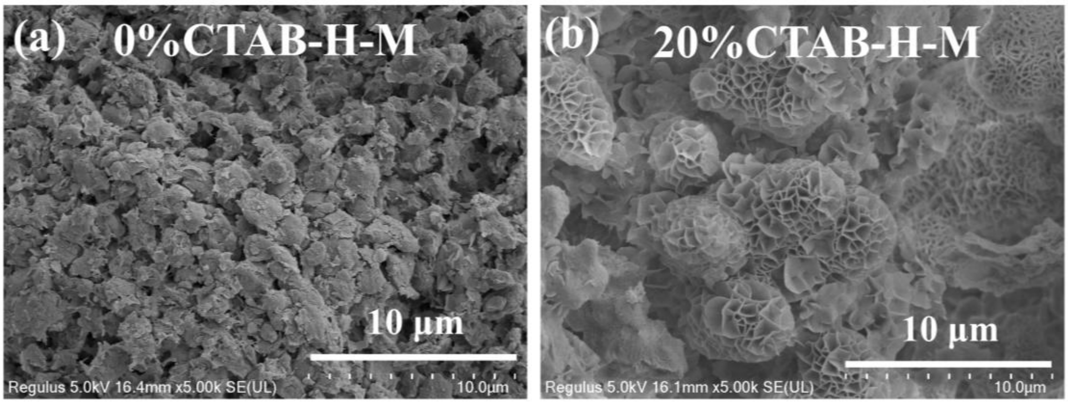

Figure S3. SEM image of 0%CTAB-H-M (**a**) and 20%CTAB-H-M synthesized by depolymerized montmorillonite geopolymer (**b**).

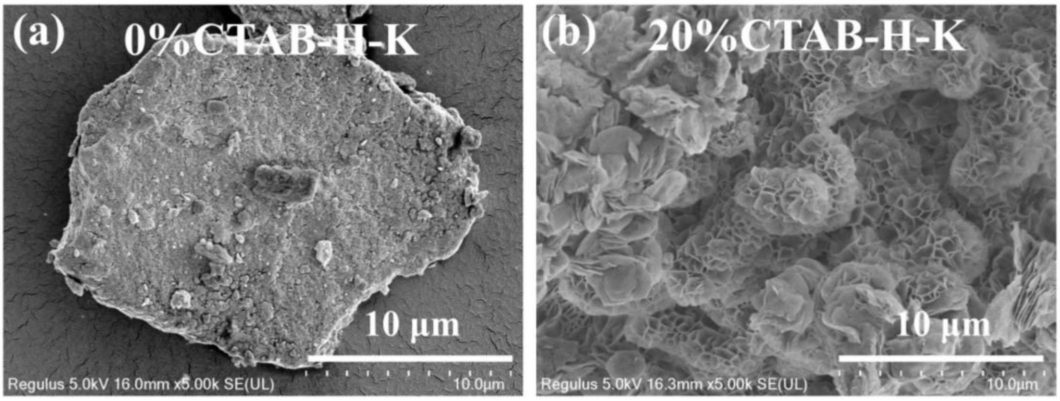

Figure S4. SEM image of 0%CTAB-H-M (**a**) and 20%CTAB-H-M synthesized by depolymerized kaolin geopolymer (**b**).
